# Evaluating differences in latent means across studies: Extending meta-analytic confirmatory factor analysis with the analysis of means

**DOI:** 10.1017/rsm.2025.10057

**Published:** 2025-12-19

**Authors:** Suzanne Jak, Mike W.-L. Cheung, Selcuk Acar, Reuben Kindred

**Affiliations:** 1 Research Institute of Child Development and Education, University of Amsterdam, Netherlands; 2 Department of Psychology, National University of Singapore, Singapore; 3 Department of Educational Psychology, University of North Texas, USA; 4 Department of Psychological Sciences, Swinburne University of Technology, Australia

**Keywords:** meta-analysis, meta-analytic structural equation modeling (MASEM), mean structures, measurement invariancestructural equation modeling (SEM)

## Abstract

Meta-analytic confirmatory factor analysis (CFA) is a type of meta-analytic structural equation modeling (MASEM) that is useful for evaluating the factor structure of measurement scales based on data from multiple studies. Modeling the factor structure is just one example of the many potentially interesting research questions. Analyzing covariance matrices allows for the evaluation of measurement properties across studies, such as whether indicators are functioning the same across studies. For example, are some indicators more indicative of the common factor in certain types of studies than in others? The additional analysis of means of the observed variables opens up many other research questions to consider such as: “Are there mean differences in mental health between clinical and non-clinical samples?” To answer such questions, it is necessary to analyze both the covariance and the mean structure of the indicators. In this paper, we present, illustrate, and evaluate a method to incorporate the means of variables in the MASEM analyses of such datasets. We focus on meta-analytic CFA, with the aim of testing differences in latent means across studies. We provide illustrations of the comparison of latent means across groups of studies using two empirical datasets, for which data and analysis scripts are provided online. The performance of the new model was tested in a small-scale simulation study. The results showed adequate performance under the tested conditions. Finally, we discuss how the proposed method relates to other analysis options such as multigroup or multilevel structural equation modeling.

## Highlights

### What is known?

- Meta-analytic structural equation modeling (MASEM) is an increasingly popular technique that enables fitting SEM models on meta-analytic data
- Meta-analytic CFA is a type of MASEM that is useful for evaluating the factor structure of measurement scales based on data from several studies
- The evaluation of between-studies differences in SEM parameters is often of interest

### What is new?

- We present and test a new method for evaluating mean structures in MASEM
- We illustrate how this enables testing differences in common factor means across studies in meta-analytic CFA
- We discuss and evaluate different levels of measurement invariance across groups of studies

### Potential impact for RSM readers outside the authors’ field?

- The availability of the method as presented in this article expands the range of research questions that can be answered with meta-analytic data
- We see many potentially interesting applications for MASEM with means in all fields

## 1 Introduction

Meta-analytic structural equation modeling (MASEM) is a meta-analytic technique to evaluate hypothesized models on the combined data of multiple independent studies.^1^ ^–^ ^3^ MASEM combines the strengths of meta-analysis (systematic synthesis of study-results) and structural equation modeling (fitting models with intricate relations between observed and latent variables). The technique is increasingly applied in very diverse fields of research such as education,^4^ psychology,^5^ environmental research,^6^ information security,^7^ medicine,^8^ and ecology.^9^

The effect sizes that need to be coded from primary studies in a MASEM study are measures of association between the variables of interest. In this article, we analyze the covariances because we ultimately wish to make comparisons across studies that involve the variances and means of the variables. MASEM then conceptually consists of two stages. First, covariance matrices from different studies are combined to form a pooled covariance matrix in a multivariate meta-analysis. Then, a structural equation model is fitted to the pooled covariance matrix. Two-stage structural equation modeling^10^ consists of these two stages, while one-stage MASEM^11^ immediately restricts the pooled covariances from the multivariate meta-analysis to a SEM model, enabling more possibilities to evaluate the influence of study-level moderating variables.

The model in a MASEM analysis can be any SEM model. Examples are path models and confirmatory factor models. Confirmatory factor analysis (CFA), whether meta-analytic or not, is a useful tool to assess the validity of measurement scales. Often the interest lies in evaluating competing measurement models.

Meta-analytic CFA is a type of MASEM that is useful for evaluating the factor structure of measurement scales based on data from several studies and is often applied to correlation matrices. For example, different studies have proposed different factor model structures for a popular measurement tool of mental well-being, the Mental Health Continuum—Short Form (MHC-SF). Iasiello et al.^12^ gathered correlations between the 14 items of the MHC-SF from 78 independent samples. Using meta-analytic CFA, they evaluated all proposed factor models on the combined data and found that the bi-factor model provided the best fit to the combined data. As another example, Acar et al.^13^ replicated and updated a meta-analytic CFA by Said-Metwaly et al.^14^ evaluating four different factor structures for the Torrance Test of Creative Thinking-Figural (TTCT-F), which consisted of five observed variables for which correlation matrices were gathered from 56 independent samples. Other examples are the evaluation of instruments for alexithymia,^15^ neuropsychological status,^16^ or implicit theories of intelligence.^17^

Modeling the factor structure is only one example of many possibly interesting research questions. Analyzing covariance matrices instead of correlation matrices allows for the evaluation of measurement properties across studies, such as whether indicators are functioning the same across studies. For example, are some indicators more indicative of the common factor in certain types of studies than in others? Analyzing additionally the means of the observed variables opens up many other research questions to consider such as: Are there mean differences in (facets of) mental health across clinical and nonclinical samples? Are there mean differences in (latent) creativity across western and nonwestern samples? To answer such questions, it is necessary to analyze both the covariance and the mean structure of the items. As Ke et al.^18^ noted recently, there currently exist no methods for simultaneously analyzing covariances and means in MASEM. In this paper, we present and illustrate a method to incorporate the means of variables in MASEM analyses. We focus on meta-analytic CFA, with the objective of testing differences in latent means across studies. In the next sections, we introduce single sample CFA, meta-analytic CFA, and the new models for the analysis of mean structures. Next, we provide illustrations of the comparison of latent means across groups of studies using two empirical datasets.

## 2 Single sample CFA

**Figure 1:**
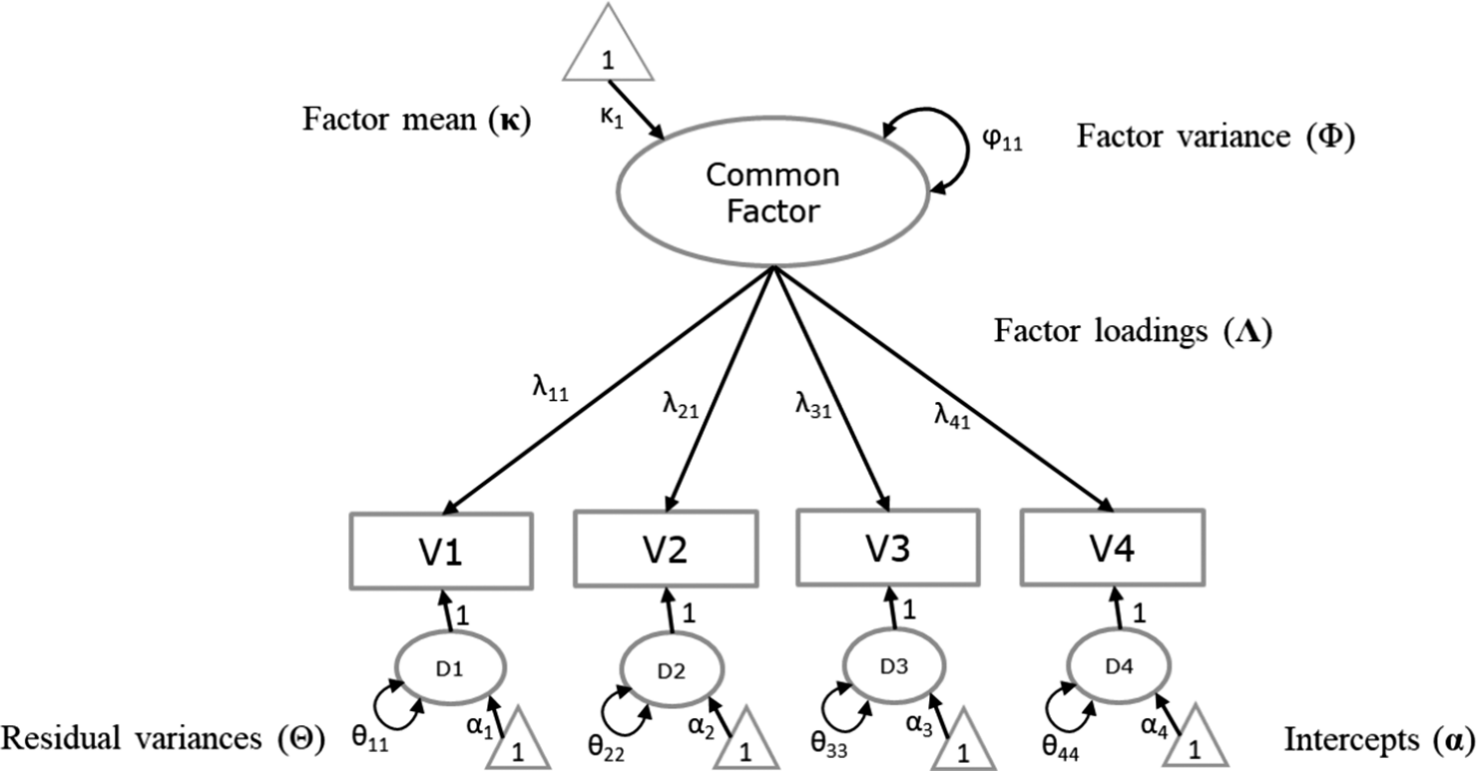
Graphical display of a one-factor model on four variables. *Note*: Observed variables are represented by rectangles. Latent variables are represented by ellipses. The small triangles represent constants of 1, depicting the mean structure. Single headed arrows indicate regression coefficients or factor loadings. Double headed arrows represent variances (or covariances). The effects of residual factors on indicators are fixed at 1 by default. The regression of the common factor on the constant of 1 (the triangle) represents the factor mean. The regressions of the residual factors (D_1_ through D_4_) on the constant of 1 represent the residual means or intercepts.

Readers who are familiar with SEM or CFA can skip this section, in which we briefly explain the CFA on a single sample. In a CFA, the covariances and means of a set of observed variables are modeled as a function of a smaller number of latent variables (common factors) that are assumed to underlie the observed variables. Suppose that a study operationalized one latent variable of interest using four observed variables. The dataset then has the observed scores for all respondents on four observed variables. A graphical display is provided in Figure 1. The common factor represents what the indicators have in common. Ideally, this corresponds to the construct that the indicators are intended to assess. The indicator-specific unique factors represent unobserved other causes of the indicators, including measurement error.

Assuming multivariate normality of the scores in the population, the mean vector and sample covariance matrix of the variables represent all relevant data. SEM then specifies a model for the covariances and the means. In case of a factor model with 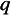 factors on 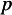 indicators, the 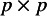 model-implied covariances 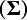 are given by:
(1)

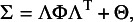

 and the 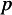 by 1 vector with model-implied means 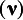 are modeled as:
(2)

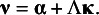



Matrix 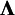 is a 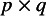 matrix containing the factor loadings, linking the common factors to the observed variables. In our example with one common factor and four observed variables, 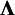 is a four by one matrix. Matrix 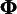 is a 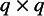 symmetric matrix with the variances (and covariances) of the latent variables. In our example, this is a one by one matrix containing the factor variance. Matrix 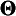 is a 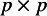 symmetric matrix containing the variances (and sometimes covariances) of the residual factors (D1 to D4 in Figure 1). In our example, there are no covariances between residual factors so 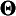 is a 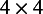 diagonal matrix. The model for the means contains two additional sets of parameters. The 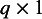 column vector 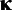 contains the means of the latent variables. In our example, this is one factor mean. The 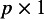 column vector 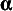 contains intercepts of the observed variables. These can also be interpreted as the means of the residual factors. The latent variables have to be provided with a scale and origin to identify the model. One way of identifying the latent variable is to fix the factor variance to 1 and to fix the factor mean to 0. This way the latent variable can be interpreted as following a standard normal distribution. Given these identification constraints on the factor mean and variance, the model matrices for the factor model in Figure 1 are given by:

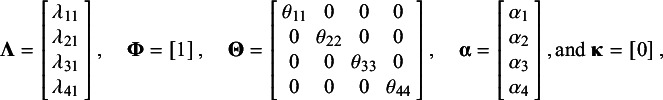

 leading to the following model-implied covariances:

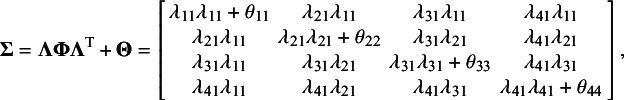

 and model-implied means: 
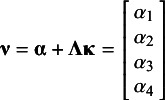
 .

The parameters of the factor model can be estimated by minimizing a discrepancy function.^19^ The CFA parameters are then estimated such that the difference between the model-implied covariance matrix 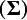 , and the observed covariance matrix, and between the model-implied means 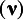 and the observed means is minimized. For more details on SEM, we refer to Bollen.^20^

## 3 Meta-analytic CFA

Meta-analytic CFA does not model the covariances and means of variables observed in one single study, but the *synthesized* covariances and *synthesized* means derived from multiple independent studies. Meta-analytic CFA with means is only applicable when the studies in the meta-analysis used comparable items, measured on equivalent scales, such that the raw scores would be comparable over studies. For fixed-effect analysis, a two-stage approach for MASEM based on covariance matrices was described by Beretvas and Furlow^21^ and Cheung and Chan.^22^ Here we consider random-effects models, allowing for between-studies heterogeneity in the study’s population covariances and means. In the next sections, we first present the general multivariate meta-analytic model that can be applied to covariances and means as effect sizes. Then, we present the new MASEM models that simultaneously evaluate the SEM structure on the average covariances and average means. Lastly, we describe the evaluation of latent mean differences across [subgroups of] studies.

### 3.1 Multivariate random-effects meta-analysis of means and covariances

In general, multivariate meta-analysis decomposes the vector 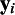 of observed effect sizes for a study 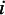 in three parts:
(3)

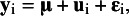

 where 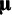 indicates the mean vector of the population effect sizes across studies, 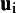 is a vector of deviations of study 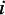 ‘s population effect size from 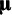 , representing the random effects, and 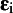 is a vector with the sampling errors of study 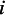 . 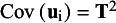 denotes the between-studies covariance matrix that has to be estimated, and 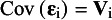 denotes the sampling covariances of the effect sizes, which are usually treated as known in a meta-analysis. When analyzing covariances as effect sizes, the dimensions of 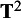 quickly increase with the number of variables of interest. For example, when evaluating a SEM model with five observed variables, there will be 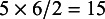 unique variances and covariances between those five observed variables as effect sizes, and 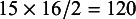 (co)variances of those effect sizes in 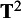 . In practice, there is almost never enough information to reliably estimate all elements in 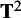 . Therefore, in MASEM of correlation or covariance matrices, the between-studies covariances are generally fixed at zero, while the variances are still estimated.^23^ Fixing the covariances at zero implies that the population effect sizes are assumed to be independent. The sampling covariances between effect sizes from the same study are still taken into account by the within-studies covariance matrices 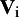 . The meta-analysis then leads to estimates of the average effect sizes across studies 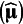 and estimates of the variances of the effect sizes across studies 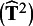 .

The same multivariate random-effects model can be applied to the variables’ means. The observed effect sizes then represent a vector of observed variable means in all studies, the 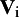 matrices contain the sampling covariances of the means in each study, and the meta-analysis will lead to estimates of the average means 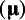 and the covariance of the means across studies 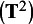 . When evaluating five observed variables in the MASEM, the dimensions of 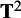 in the model for the means will be five by five, which is much smaller than the dimensions of this matrix for the covariances. It is therefore more likely that the between-studies covariances can be estimated as well, so that 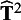 is not diagonal but symmetric. In the rest of the section, we consider multivariate meta-analysis of covariances as well as multivariate meta-analysis of means. To distinguish these, we use subscripts to indicate the type of effect sizes: 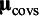 and 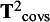 _s_ denote respectively the averages and (co)variances of *covariances* across studies, and: 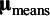 and 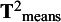 denote respectively the averages and (co)variances of *means* across studies.

The meta-analysis of means and covariances can also be combined in one multivariate model. The total mean vector 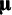 is then a concatenated vector of 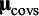 and 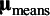 , while the total 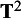 consists of 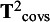 and 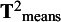 plus the between-studies covariances of the means and the covariances ( 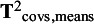 ) of which the lower triangular is:

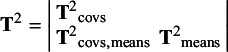



The study-specific sampling covariance matrices 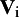 are constructed using the same structure:

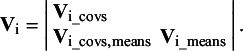



### 3.2 Fitting meta-analytic factor models on means and covariances

Currently, there exist no methods for the evaluation of meta-analytic CFA with latent means. Therefore, we propose a new model that extends the multivariate meta-analysis of covariances and means by restricting the average effect sizes to the model-implied moments of a confirmatory factor model. The observed effect sizes consist of the (vectorized) covariance matrices of the observed variables in each study, and a vector of means of the observed variables for each study. The multivariate meta-analysis model is applied to the observed effect sizes. In addition to modeling the meta-analytic covariances, we propose one-stage MASEM^11^ on covariances and means:
(4)

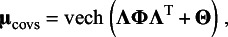



(5)

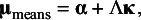

 where with *p* observed variables and 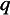 common factors, 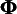 denotes the 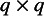 covariance matrix of common factors, 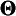 denotes the 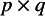 (diagonal) covariance matrix of residual factors, and 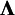 is the 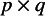 factor loading matrix that regresses the observed variables on the common factors. Each common factor must be provided a scale, which is often done by fixing the common factor variances to one. The vech()-operator provides the half vectorization of the model-implied covariance matrix, which leads to a vector of model-implied covariances of the same dimensions as 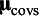 . The vector 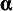 represents a 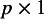 column vector of measurement intercepts, 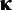 is a 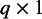 column vector of common factor means. In order to provide an origin to the common factors, either the factor means or one of the intercepts has to be fixed, commonly to zero.

The meta-analytic CFA models for the covariances and the means can be fit simultaneously using the metaSEM package,^24^ which uses the OpenMx package^25^ as the backend in the R statistical platform.^26^ For the specific implementation, we refer to the R-scripts in the supplementary materials. A test statistic of the hypothesized factor model can be obtained by performing a likelihood ratio test with a saturated covariance and means model.^11^

Fixing the factor means ( 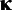 ) to zero will lead to estimates of 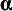 that are identical to 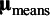 , because the mean structure is saturated. It becomes more interesting when fitting meta-analytic CFA models in which the latent means are allowed to differ across studies. We discuss this in the next sections.

## 4 Evaluating latent mean differences across studies

Suppose that one obtained the sample covariances and sample means of some measurement instrument from a large number of primary studies. Some of these studies focused on clinical populations, and some of them evaluated nonclinical populations. A research question could be whether the latent means differ across the clinical and nonclinical populations. An important prerequisite before comparing latent means across groups is to establish a sufficient level of *measurement invariance* across groups, meaning that the measurement of the construct of interest should be the same across groups in order to make valid comparisons on the construct across groups.^27^ Three increasingly restrictive levels of invariance are called *configural invariance*,^28^ representing equal factor structures across groups, *weak factorial invariance*,^29^ representing equal values of factor loadings across groups, and *strong factorial invariance*,^30^ representing equal values of factor loadings and intercepts across groups. The means of common factors can only be compared across the groups if strong factorial invariance across groups holds to a sufficient degree.^30^ ^,^ ^31^ It is therefore essential to test whether strong factorial invariance holds before making comparisons on latent means. In practice, this condition often does not hold, so that violations of invariance should be taken into account.^32^ The process of evaluating measurement invariance and taking violations into account is shown in the illustration in the next section. First we explain the different levels of measurement invariance.

We can formulate the three levels of invariance using the following equations. With configural invariance across subgroups of studies, the same factor model structure is applied to all groups, but all parameters in the meta-analytic factor model are allowed to be different across the subgroups, as indicated by adding the subscript 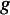 :
(6)

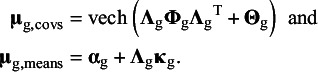



For identification, the factor variances in 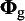 would be fixed at one for both groups, and 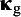 would be fixed at zero for both groups.

Weak factorial invariance across groups of studies entails equal factor loadings across groups of studies:
(7)

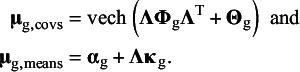



In a model with weak factorial invariance where scaling is applied by fixing common factor variances to one, the scaling constraints only need to be applied in one group. That is, common factor variances can (and should) be freely estimated in all other groups.

Strong factorial invariance holds if, in addition to equal factor loadings, the intercepts are equal across groups:
(8)

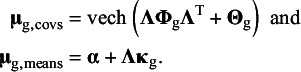



With strong factorial invariance, the group differences in variables’ covariances are a function of differences in latent (co)variances 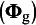 and residual (co)variances 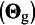 , and group differences in variable means are a function of group differences in common factor means 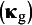 . When providing origin through fixing factor means, the factor means need to be fixed at zero in one group and can be freely estimated in the other group(s). The estimated factor means then represent the difference in factor means across groups. Table 1 provides an overview of the different levels of measurement invariance and the associated restrictions and interpretations.

**Table 1:** Overview of three levels of measurement invariance in a CFA and associated properties

			Comparisons on
Level of	Restrictions on		common factors
invariance	parameters	Interpretation	allowed
Configural invariance	No equality restriction on parameters	The same factor structure applies	None
Weak factorial invariance	Equal factor loadings	The observed variables are equally indicative of the common factor	Comparison of common factor variances
Strong factorial invariance	Equal factor loadings and intercepts	The observed variables are equally indicative, and equally “difficult.” Observed mean differences are the result of mean differences in the common factor.	Comparison of common factor variances and common factor means

The models above describe conditions in which all factor loadings and intercepts are equal. In practice, one may also find that some measurement parameters are equal across groups, and some are not. This is referred to as *partial invariance*.^33^ Ideally, the majority of the indicators have invariant factor loadings and intercepts for an unbiased comparison of factor means across groups.^34^

There exist multiple ways of evaluating measurement invariance across groups. One option is to fit the model to separate datasets of the groups and test the equality of factor loadings and intercepts by constraining those parameters to be equal across groups, and evaluating the difference in model fit with an unconstrained model. This is called the multigroup approach.^30^ An advantage of multigroup modeling is that it is straightforward to allow all parameters to differ across groups. In other words, all parameters can in principle be moderated by group membership. A limitation of the approach is however that the approach is only applicable with grouping variables as moderators, and that the number of studies in each group is a subset of the total number of studies.

A more flexible way of testing measurement invariance in meta-analytic SEM is to introduce group membership as a study-level moderator in the model, as is possible in one-stage MASEM.^11^ Here we consider the situation of wanting to make comparisons across two groups. The grouping variable would then be a dummy variable indicating whether the study is in the reference group (value 0) or the other group (value 1). For comparing more than two groups, one would need to add more dummy variables. Parameters that are allowed to be different across groups can be regressed on the moderator, and parameters that should be equal across groups are not regressed on the dummy moderator. So, in a model with configural invariance across groups, all CFA parameters are moderated by the dummy variable indicating group membership. In the model with strong factorial invariance, the factor loadings and intercepts are not regressed on the dummy variable, while the factor means, factor variances, and residual variances are. This approach is called moderated nonlinear factor analysis and is very suitable for evaluating measurement invariance in primary data (MNLFA).^35^ ^,^ ^36^ However, the technique is broadly applicable, for example to integrate datasets in IPD-meta-analyses.^37^ In the rest of the article, we will refer to this approach as the “regression approach.”

In the regression approach, each parameter that is regressed on a moderator will be decomposed into an intercept of that parameter, and the regression coefficient representing how a one point increase in the moderator variable modifies the parameter. For example, if one lets a factor loading be a function of the study-level variable 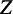 , the factor loading 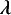 gets a subscript g, to indicate that it varies with studies’ values on 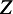 : 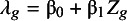 . The parameters that will be estimated are 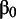 _,_ representing the intercept of the factor loading (the expected factor loading if 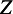 equals zero), and 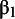 _,_ representing the linear effect of 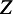 on the factor loading. If 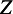 is a dummy variable, then 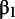 reflects the group difference in the factor loading. If 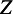 is a continuous variable, 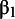 shows how the factor loading is expected to change with one unit increase in 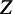 .

The regression approach can fit any model that the multigroup approach can, but it is much more general. The biggest advantage of this regression approach is that it is not only possible with grouping variables as moderators, but also with continuous moderators. This would allow one to evaluate strong factorial invariance and latent mean differences across values of a continuous study-level variable such as mean age of participants, proportion of females in the sample, or some continuous operationalization of study quality. Moreover, the regression approach allows the evaluation of multiple moderators (continuous or dichotomous) at the same time.

## 5 Illustrations

We illustrate testing latent mean differences and strong factorial invariance using two examples. The first example involves data on post-traumatic stress disorder. The second example uses data obtained from a measurement instrument for creativity. The modeling procedures involve fitting the factor structure to the total dataset, evaluating whether a sufficient degree of measurement invariance holds across groups of interest, and comparing latent means across groups of studies. The data and analysis scripts are available from OSF (https://osf.io/wzg7s). In order to facilitate future analyses, we provide detailed explanation of the syntax used to fit a strong factorial invariance model using the R-package metaSEM^24^ in Supplementary Appendix A.

## 6 Illustration 1—International Trauma Questionnaire

**Figure 2:**
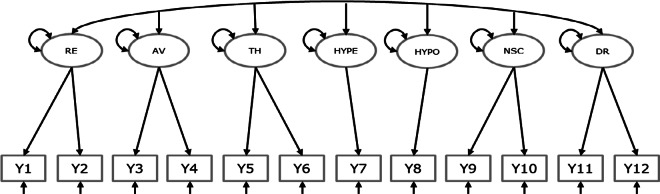
Hypothesized seven-factor model in Illustration 1. *Note*: Graphical displays of the mean structure and the residual factors are omitted. All seven factors covary. RE = re-experiencing the event, AV = avoidance of reminders of the event, TH = a sense of threat, HYPE = affective hyperactivation, HYPO = affective hypoactivation, NSC = negative self-concept, DR = disturbance in relationships. For identification, all factor variances are fixed at 1. To identify the two single indicator factors, the residual variances of Y7 and Y8 are fixed at zero. All factor loadings are freely estimated.

Kindred et al.^38^ gathered covariance matrices and means of the items of the International Trauma Questionnaire (ITQ). The ITQ was developed by Cloitre et al.^39^ to assess Post-Traumatic Stress Disorder (PTSD) and Complex PTSD (CPTSD) according to the criteria outlined in the eleventh iteration of the International Classification of Diseases (ICD-11) by the World Health Organization. Kindred et al.^38^ evaluated the factor structure of the ITQ based on the correlation matrices of 56 studies and found that the seven-factor model depicted in Figure 2 fitted the meta-analytic data well. The seven factors represent: re-experiencing the event (RE), avoidance of reminders of the event (AV), a sense of threat (TH), affective hyperactivation (HYPE), affective hypoactivation (HYPO), negative self-concept (NSC), and disturbance in relationships (DR). RE, AV, TH, NSC, and DR are each operationalized by two items, while HYPE and HYPO are single-indicator factors, so the total scale consists of 12 items. Each item was scored on a 5-point Likert scale, which we treat as continuous in the analyses. In addition to gathering inter-item correlations, the authors also gathered the item’s standard deviations and means, and several study-level characteristics. We use these to illustrate testing differences in the latent means across studies based on clinical samples (7 out of 56) versus studies based on nonclinical samples (49 out of 56). We expected that the clinical samples scored higher on all seven common factors reflecting CPTSD.

### 6.1 Analysis

As a baseline model, we applied a model with a saturated mean and covariance structure to the total sample of studies. This provides estimates of the pooled covariances and pooled means across studies. Then, we fit the factor model to the data of the total sample. We evaluated model fit using the chi-square statistic, RMSEA, and use the AIC and chi-square difference test for model comparisons. The chi-square statistic is obtained by taking the difference in −2 loglikelihoods of the factor model and a saturated model. Next, we fit several models in which certain parameters are regressed on a dummy variable indicating whether a study evaluated a clinical or nonclinical sample. As a baseline model to be able to evaluate the model fit, we estimate a saturated model in which all variances and covariances are moderated. Next, we fit models representing configural invariance, weak factorial invariance, and strong factorial invariance across groups of studies. In the configural invariance model, the factor structure is the same across groups, but the parameters can have different values over groups. In this model, all free CFA parameters were regressed on the dummy variable, the factor variances and means were respectively fixed at 1 and 0 for scaling. In the weak factorial invariance model, we constrain the factor loadings to be equal across groups of studies. In this model, the factor variances were moderated by the dummy variable to allow for differences in heterogeneity of factor scores across groups, while the intercept value of the factor variances were fixed at 1. In the strong factorial invariance model, the intercepts were also constrained to be equal across groups. The factor means were then moderated by the dummy, with the intercept of the factor mean fixed to zero to provide origins to the common factors. The effect of the dummy then represents the mean difference in factor means across groups (and the difference in observed means for the two single indicator factors). This way, the estimates of the factor means in the nonclinical group represent the factor mean differences across groups of studies. We inspected the chi-square difference and the difference in AIC between the MASEM models with and without the invariance constraints on the intercepts and factor loadings to evaluate whether the constraints were tenable. For testing statistical significance, we used a nominal alpha level of 5% throughout.

### 6.2 Results

The fit of the 7-factor model on the overall dataset was satisfactory 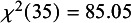 , 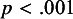 , 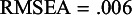 . Fitting either the saturated moderated model, or the model with configural invariance across sample type (0 = nonclinical, 1 = clinical) did not lead to a converged solution. This is not very surprising given the small number of studies relative to the number of parameters to be estimated, and the large number of moderation effects. The models with weak factorial invariance and strong factorial invariance are more constrained and resulted in a converged solution. Without the likelihood of the saturated model, we cannot evaluate the overall fit of these models. However, we can compare the fit of the weak invariance model with the fit of the strong invariance model using the likelihood ratio test and the AIC. The likelihood ratio test indicated that the strong invariance model fitted significantly worse than the weak invariance model ( 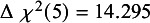 , 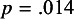 ) ^1^, and the AIC was lower for the weak invariance model (AIC = −1400.568) than for the strong invariance model (−1396.274). Investigating partial strong invariance was not possible in this model with only one or two indicators per factor.^33^ We therefore conclude that strong factorial invariance across clinical and nonclinical samples did not hold, so factor means cannot be validly compared across samples.

This illustration used data on 12 observed variables and 7 latent factors, which led to a large number of CFA parameters to be estimated and possibly moderated. Moreover, with 12 observed variables there will be 12 means of variables, and 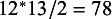 covariances among those variables. So even with a diagonal heterogeneity matrix on the covariances ( 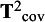 ), there were 78 between-studies variances of the covariances and 78 between-studies covariances of the means (symmetric 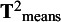 ) to be estimated. The next example uses only five observed variables, which lowers the computational burden substantially. With five observed variables, there are five means, and 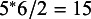 covariances among those variables. A diagonal 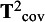 then has 15 variances to be estimated, while a symmetric 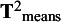 would have 5 between-studies variances of means, and 10 between-studies covariances of means.

## 7 Illustration 2—Torrance Tests of Creative Thinking-Figural

The TTCT-F is a test in which participants are asked to create drawings based on visual prompts. The test includes three activities: Activity 1: Picture Construction, Activity 2: Picture Completion, and Activity 3: Lines or Circles. These activities are scored based on five criteria: fluency, originality, elaboration, abstractness of titles, and resistance to premature closure. Fluency measures participants’ ideational productivity, which is the count of the relevant, meaningful drawings they produce. Originality is the sum of unusual and infrequent responses produced across the three activities, with additional points awarded for drawings that incorporate more than a single visual prompt. Elaboration reflects the amount of detail and sophistication in the responses, where any additions beyond a basic descriptive figure earn elaboration points. Abstractness of Titles focuses on the level of abstraction in the titles generated for the drawings; descriptive or abstract titles earn points, while basic titles do not receive any points. Finally, Resistance to Premature Closure indicates openness and the suspension of judgement, measured by a count of drawings with either extended closure or no closure at all.

The data in this illustration are the covariance matrices and mean vectors of 38 independent samples on the TTCT-F, which represent an extension of the data obtained by Acar et al.^40^ We apply the two-factor structure that was found most appropriate in earlier research.^13^ ^,^ ^14^ ^,^ ^40^ It contains an Innovation factor with the first two subtests as indicators, and an Adaptation factor that explains the covariances between the last three subtests.

### 7.1 Analysis

We followed the same analysis strategy as in the previous example. First, we evaluated the overall factor structure of the TTCT-F on these data by comparing the fit of the CFA with the fit of a saturated SEM structure. Next, factor mean differences across western and nonwestern samples were evaluated by testing measurement invariance with the regression approach. There were 8 nonwestern samples and 30 western samples.

### 7.2 Results

**Figure 3:**
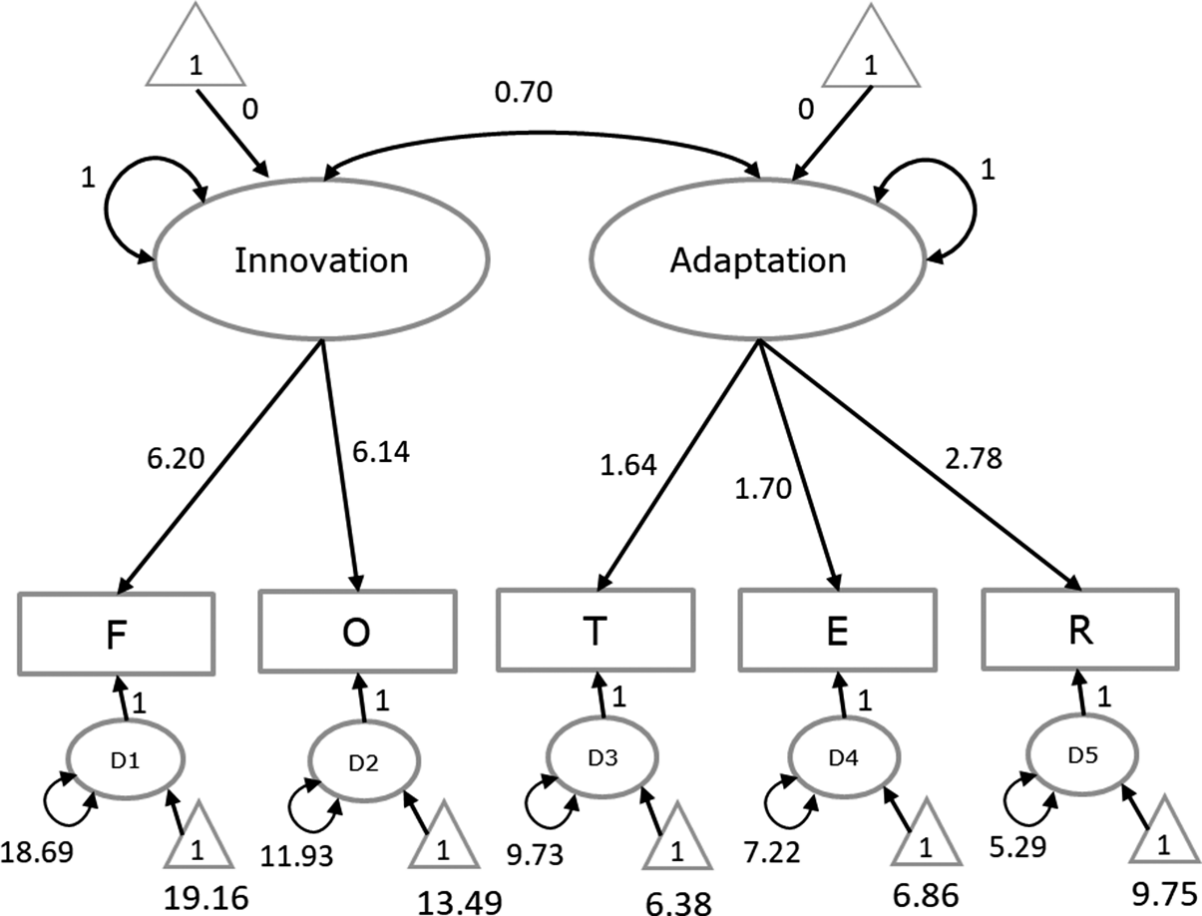
Parameter estimates of fitting the hypothesized meta-analytic CFA to the total set of samples in Illustration 2. *Note*: F = Fluency, O = Originality, T = Abstractness of Titles, E = Elaboration, R = Resistance to Premature Closure.

**Overall fit.** The hypothesized factor model fitted the data well, 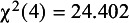 , 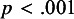 , 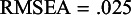 . A graphical display of the fitted model with parameter estimates is given in Figure 3. Note that the two factor means are fixed at zero in this model. As a result, the estimates of the five intercepts of the indicators are equal to the pooled means of the five variables as they would be obtained using a multivariate meta-analysis of the means without fitting the CFA. The more interesting results can be obtained by making comparisons of means across western and nonwestern samples.

**Testing differences in latent means.** Next, we fit a model with configural invariance across nonwestern and western samples. In the configural model, all estimated values of the CFA parameters are allowed to be different across the western and nonwestern studies, except for the factor variances (fixed at 1) and factor means (fixed at 0). The fit of this model was satisfactory and is reported in Table 1. Next, we constrained the factor loadings to be equal across samples by removing the moderating effect of the dummy variable on the factor loadings, representing weak factorial invariance in the meta-analytic CFA. In this model, the factor variances were fixed at 1 for the western group, and the effect of the moderator represented how the estimate in the nonwestern group differed from 1. The weak invariance model has three more degrees of freedom than the model with configural invariance (five moderating effects on factor loadings are removed, two moderating effects on factor variances are added). The fit of the model with weak invariance was significantly worse than the fit of the configural model, indicating that not all factor loadings can be considered equal across the subgroup of western and nonwestern samples. Next, we fitted five separate models with partial weak invariance, where in each model one of the factor loadings was not constrained to be equal across groups. The model in which the factor loading of variable Elaboration (E) was not constrained across groups led to the best model fit. The fit of this model was not significantly worse than the fit of the configural invariance model (see Table 2). The estimated factor loading for the western samples was 1.391, and the moderating effect of the dummy was 1.673, indicating that the estimated factor loading is 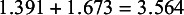 for nonwestern samples.

**Table 2:** Fit statistics for the overall factor model and the invariance models of Illustration 2

Model	df	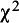	p	AIC	Models	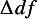	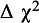	p
1.Overall	4	24.402	<.001	4926.416				
**Invariance models**								
2. Configural invariance	8	25.29	.001	4887.919				
						2 versus 3	3	20.407	<.001
3. Weak invariance	11	45.698	<.001	4902.326				
						2 versus 4	2	1.054	.590
4. Partial weak invariance	10	26.346	.003	4884.974				
						5 versus 4	2	0.428	.807
5. Partial strong invariance	12	26.774	.008	4881.402				

Next, we fit a model with partial strong factorial invariance, in which in addition to the four invariant factor loadings, the four intercepts were held invariant across groups. This model did not fit significantly worse than the model with partial weak invariance. The estimated differences in factor means were 0.445 for the Innovation factor and − 1.168 for the Adaptation factor, indicating higher Innovation and lower Adaptation in nonwestern samples. Both estimates differed significantly from zero, indicating statistically significant differences in factor means across groups. These estimates can be standardized in order to interpret them as standardized mean differences (SMDs). Dividing the raw mean difference by the pooled standard deviation of the common factor across the two groups provides a standardized mean difference. For Innovation, the SMD equals 0.359, and for Adaptation the SMD equals −1.287.

## 8 Simulation study

In order to evaluate the performance of the new method, we conducted a small simulation study. This study serves as a proof-of-concept and provides a first impression of the quality of the outcomes of MASEM with means. We provide the syntax and results of the simulation study on OSF.

## 9 Data generating model and manipulated factors

**Figure 4:**
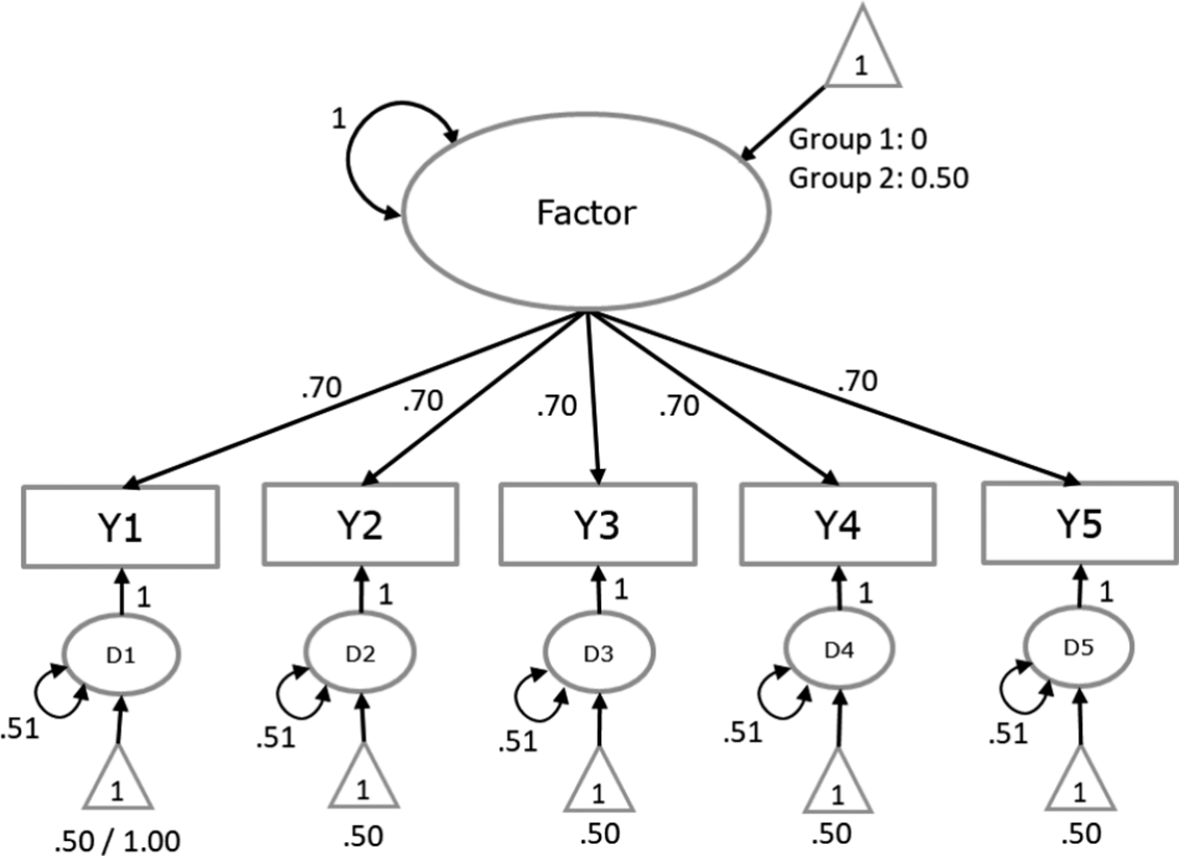
Data-generating model with population values leading to the model-implied covariance and mean vectors for the simulation study. *Note*: In condition where strong factorial invariance holds, the intercepts were .50 in both groups. In conditions where strong factorial invariance did not hold, the intercept of the first indicator was 0.50 in Group 1 and 1.00 in Group 2.

We generated data based on a one-factor model with five indicators in two groups of studies (Figure 4). For both groups of studies, all factor loadings were .70, all residual variances were .51, and all intercepts were .50 in conditions with strong factorial invariance. The common factor mean was set to zero in Group 1 and 0.50 in Group 2. These population values lead to a model-implied covariance and mean vector for both groups. Heterogeneity was introduced by specifying between-studies variances for all covariances of .01, between-studies variances for all means of .20, and between-studies covariances of .10 between the means .

We generated data in conditions where strong factorial invariance held (all intercepts and factor loadings are equal across groups), and where strong factorial invariance did not hold. In the latter cases, the intercept of the first variable was 1.00 instead of .50 in Group 2. Moreover, we evaluated conditions with 15, 20, or 24 studies per group (so 30, 40 or 50 studies in total). Combining these factors leads to six conditions, for which we generated 1000 meta-analytic datasets each.

## 10 Fitted models and evaluation criteria

To each generated dataset we the fitted four models: (1) saturated model, (2) configural invariance model (10 dfs), (3) weak factorial invariance model (14 dfs), and (4) strong factorial invariance model (18 dfs). We evaluated the following outcomes:

1. Estimation bias in the common factor mean in Group 2
2. Whether strong factorial invariance was rejected based on the overall 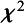 -test
3. Whether the 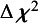 -test rejected strong factorial invariance in favor of weak factorial invariance 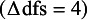

4. Whether the 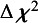 -test rejected weak factorial invariance in favor of configural factorial invariance 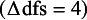

5. Which of the four models was selected based on the AIC (which model has the lowest AIC-value?)
6. Which of the four models was selected based on the BIC (which model has the lowest BIC-value?)

The common factor mean can only be evaluated in the model with strong factorial invariance, and should be well estimated (close to .50) in conditions where strong factorial invariance is the data-generating model. Ideally, the AIC and BIC should select the data-generating model. The 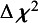 -test should reject strong factorial invariance in conditions where it does not hold, and should not reject weak factorial invariance more frequently than the employed significance level of 5% in any of the conditions. The overall 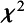 -value should be statistically significant in conditions where the true model is weak factorial invariance, and is expected to be statistically significant in 5% of the replications if strong factorial invariance is the true model.

## 11 Results

The outcomes of the simulation study are reported in Table 3. All models converged in all replications. In the conditions where strong factorial invariance holds in the population, the estimated difference in factor means between groups of studies was very close to the true value of .500 on average. The largest absolute bias found was .008. If strong factorial invariance was not the true model, the estimated factor means were overestimated in all conditions, with absolute bias between .146 and .150. These findings show the importance of testing whether strong factorial invariance actually holds before interpreting the factor mean differences.

**Table 3:** Average of the estimated factor means, rejection rates of 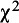 -tests, and selection rates of the AIC and BIC under conditions with strong or weak invariance as the data generating model, and varying numbers of studies

True		Factor				AIC selected		BIC selected	
model	K	mean	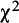 RR	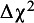 RR		model		model	
				Strong	Weak				
			Strong	versus Weak	versus Conf	Strong	Weak	Strong	Weak
Strong	30	.505	.086	.072	.063	.839	.110	1.00	.000
	40	.492	.059	.072	.044	.839	.130	1.00	.000
	50	.508	.081	.052	.066	.863	.087	1.00	.000
Weak	30	.650	.656	.884	.071	.060	.810	.983	.017
	40	.646	.738	.945	.046	.027	.862	.963	.037
	50	.646	.848	.984	.075	.011	.858	.905	.095

*Note:* Strong = Strong factorial invariance model, Weak = Weak factorial invariance model, Conf = Configural invariance model, k = number of studies in total, Factor mean = average estimate of the factor mean in the strong factorial invariance model over 1000 replications, 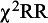 = proportion of replications for which the strong invariance model was rejected by the overall 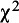 -test, 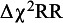 = proportion of replications for which the more restricted model was rejected by the 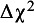 -test, AIC/BIC selected model = proportion of replications for which a model had the lowest AIC/BIC.

In the conditions where strong factorial invariance did not hold, the overall 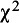 -test correctly rejected strong invariance in .656 of the replications with 30 studies, and increased to .738 and .848 of the replications with 40 and 50 studies, respectively. In the conditions where strong invariance was the true model, the false positive rates of the overall 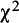 -test were a bit higher than the expected .050, with values of .086, .059, and .081. The 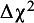 -test of rejecting strong invariance in favor of weak invariance had more power than the overall test, leading to .884 of the replications with 30 studies, and increased with number of studies. In the conditions where strong invariance was true, this 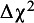 -test incorrectly rejected strong invariance in .044 to .066 of the replications. False positive rates of rejecting weak invariance in favor of configural invariance ranged from .044 to .075 across all conditions.

The AIC selected the correct model in more than 80% of the replications in all conditions. The BIC selected the strong invariance model in more than 90% of the replications, even in the conditions where weak invariance was the correct model.

## 12 Discussion

In this article, we presented a method for integrating the analyses of means in MASEM. Although MASEM already exists for over 30 years, the illustrations in this article present the first MASEM analyses that investigated common factor means. The evaluation of differences of factor means across groups of studies requires the evaluation of measurement invariance across the groups of studies. MASEM with latent means enables testing the increasingly restrictive invariance models, with strong factorial invariance being the one that allows valid comparisons in factor means.

In the first illustration, we were not able to estimate all models that were relevant for evaluating measurement invariance. This is an example of what researchers may encounter in practice when they try to evaluate MASEM models with small numbers of studies relative to the number of observed variables. The second example used only five observed variables and did not lead to problems estimating the models.

A small-scale simulation study showed that the differences in factor means across groups of studies can be well estimated if strong factorial invariance indeed holds and that the estimates will be biased if strong factorial invariance is incorrectly assumed. For testing whether strong factorial invariance holds, the overall 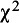 and 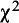 -difference test worked reasonably well, although the false positive rates were slightly higher than expected. The BIC is not recommended to select models as this index did not differentiate between the weak and strong invariance models. The AIC worked quite well in selecting the correct model.

## 13 Extensions and alternative approaches

The necessity of evaluating of measurement invariance in meta-analytic CFA before comparing latent means across groups may come across as inconvenient or a disadvantage of the approach. For example, one may find that strong factorial invariance across groups does not hold , preventing a valid comparison of latent means across groups. A much easier approach seems to be comparing the observed scale means or sum scores of the indicators across groups instead. However, such an analysis assumes that the measurement is invariant across groups, leading to biased mean comparisons if invariance does not hold.^32^ It is therefore actually an advantage that testing measurement invariance becomes possible when having study-specific data on the item level, so that one can evaluate differences in latent means instead of observed means. Moreover, observed scale means are affected by measurement error while latent means are not, so that corrections for unreliability are not needed in meta-analytic CFA.^41^

The two datasets in our illustrations consisted of item scores of established measurement instruments, resulting in data on exactly the same items scored in equivalent ways in the different studies. There may also be the situation in which the relevant studies did not use the exact same measurement instruments for operationalizing the construct of interest. In such cases, the applicability of MASEM with means depends on the comparability of the scores across studies. If the raw datasets of the studies are available, they may be harmonized to make the observed score comparable.^42^ If the studies only provide summary statistics (covariances and means) the meta-analyst has to make a judgment of the comparability of the scores based on the information provided on the used items and response scales in each specific study.

In our illustrations, we focused on testing latent means across *groups* of studies, but the regression approach is more general. One could also test latent mean differences across values of a continuous study-level variable by regressing the relevant parameters on the continuous variable. We provided an example analysis concerning the proportion of females in the sample on the TTCT-F data on the OSF page. Moreover, it is possible to evaluate measurement invariance using multiple moderators simultaneously. Increasing the between-studies model logically necessitates increasing the amount of between-studies information. In real datasets, the number of available studies may not be sufficient to evaluate multiple moderators at the same time.

We fitted models representing different levels of invariance across levels of the dummy variables by regressing the CFA parameters on the dummy variable. The between-studies covariance matrix 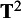 was not moderated in our examples, indicating that the amount of between-studies heterogeneity in means and covariances was assumed to be equal across values of the study-level moderator. This assumption can be relaxed, i.e., one could also fit models in which the heterogeneity parameters are moderated by a study-level moderator. Such an analysis may not often be feasible in practice, because the number of parameters to be moderated will become too large.^43^ In our examples, such models indeed did not lead to a converged solution.

Structural equation models can also be fit to the observed mean vector and covariance matrix of a single sample. Therefore, one could be tempted to fit the CFA to the covariances and means of the individual studies and meta-analyze the SEM parameters. However, with such an approach, the latent means are not identified in the individual studies, so means cannot be compared. Alternatively, one could fit a large multigroup model on the covariances and means of all studies simultaneously and apply strong invariance constraints across all groups to estimate factor means in all but one group. The factor means could then be meta-analyzed and predicted by study-characteristics. Conceptually, the invariance restrictions in a large multigroup model are stricter than in the meta-analytic CFA. That is, in the multigroup model, exact equality of factor loadings and intercepts across all studies is required, while in the meta-analytic CFA model the equality constraints are applied to a model for the average covariances and average means per subgroup. For the other parameters (factor variances and covariances, residual (co)variances), the multigroup model is more flexible because each study has its own specific estimate, whereas in the meta-analytic CFA study, differences in these parameters are only reflected by the between-studies heterogeneity parameters 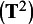 .

## 14 Directions for future research

Testing differences in factor means across groups of studies, and consequently testing strong factorial invariance across groups of studies, has strong resemblance with testing measurement invariance across groups of clusters in two-level SEM. Muthén et al.^44^ and Ryu^45^ consider examples concerning data from individual students nested in schools. Measurement invariance across a group of public schools and a group of catholic schools is then evaluated by fitting two-level factor models to both groups, and evaluating the equality of the relevant parameters across the groups of schools. In such an analysis, one basically tests measurement invariance based on the averages within the public schools and the averages within the catholic schools. This is different from testing measurement invariance across *all* schools, which requires fitting a model with additional constraints.^46^ Similarly, when testing invariance using MASEM with means, the random-effects model will allow for the remaining heterogeneity in the covariances and means by estimating the 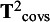 and 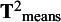 matrices in the groups of studies. Determining the exact similarities and differences across MASEM with means (on summary statistics) and two-level SEM (on raw data) is an interesting avenue for further research.

MASEM with means could also be interesting in cases where there is no interest in comparing means across studies, but in comparing means within the studies. When meta-analyzing studies that used the same measurement instrument at two timepoints, one could fit a meta-analytic longitudinal CFA to the studies’ average covariances and means. By applying invariance constraints on the factor loadings and intercepts across timepoints within studies, the factor means at all but one timepoint can be freely estimated, allowing for the evaluation of change in latent means over time.^47^ If the individual studies differ in the lag between timepoints, this can be accounted for by including lag as a study-level moderator affecting the parameters that may be affected by lag. The availability of the method as presented in this article may motivate meta-analysts to gather the data necessary to carry out such an analysis.

We see many potentially interesting applications for MASEM with means, and we showed that the technique is applicable and leads to interpretable results on empirical datasets. We also provided some initial insights into the performance in a limited number of conditions. In order to evaluate how the technique performs under different model and data conditions, a more extensive simulation study would be needed. Interesting factors to consider in such a simulation study would be the size of the model (number of parameters to be estimated) relative to the number of studies and the individual sample sizes, the size of the non-invariance, and the size of the factor mean differences.

## Supporting information

Jak et al. supplementary material Jak et al. supplementary material

## Data Availability

The data and syntax to replicate the analyses presented in this article are openly available from OSF at https://osf.io/wzg7s.

## Footnotes

1
The difference in number of estimated parameters is 5 because in the strong invariance model the moderating effects on the 12 intercepts are removed, while moderating effects on the 7 factor means are added (12–7 = 5).

## Supplementary material

To view supplementary material for this article, please visit http://doi.org/10.1017/rsm.2025.10057.
