# Supplementary material for: Evaluating differences in latent means across studies: Extending meta-analytic confirmatory factor analysis with the analysis of means
Source: Res Synth Methods. 2025 Dec 19;17(3):498–516. doi: 10.1017/rsm.2025.10057 (PMC13126220; doi:10.1017/rsm.2025.10057)
Supplement: Jak et al. supplementary material [file S1759287925100574sup001.pdf]

Appendix A Fitting a model with strong factorial invariance across two groups of studies: Explanation of the R-syntax

This appendix clarifies the organization of the data needed for applying CFA with means in two groups of studies, and explains the R-syntax used to specify the models using the metaSEM package. Below, we annotated the syntax of fitting the strong factorial invariance model to the data of the TTCT-F.

```
library(metaSEM)
```

Firs one needs to load the metaSEM package. Next, the `Cor2DataFrame()` function prepares the data for analysis and needs as arguments:

A list of covariance matrices of the samples: `data_acar$covs`

A vector of sample sizes: `data_acar$n`

A matrix with k rows containing the variable means: `meandata`

```
df <- Cor2DataFrame(data_acar$covs,n=data_acar$n,Means=meandata,
cor.analysis=FALSE)
```

The moderator variable can be added to the '`data`' slot of `df`. Moderators should be numeric variables. For '`culture`' we used 0 for Western samples and 1 for non-Western samples:

```
df$data$NonWestern <- ifelse(data_acar$culture=='Western', yes=0,
no=1)
```

The model can be specified using lavaan syntax. Factor loadings are specified using the '`=~`' operator, connecting the common factor at the left side to the indicator name(s) at the right side. Variances and covariances are specified using the '`~~`' operator with the names of the involved variables on each side. Simple regression is specified using the '`~`' operator. Means and intercepts are operationalized as variables regressed on a constant of one. The first line of the syntax:

```
Innov =~ L1*F + L2*O
```

Thus specifies that the latent variable ‘`Innov`’ has two indicators that have the names ‘F’ and ‘O’. The two factor loadings are provided labels by including ‘`label*`’ in front of the variable name. The factor loadings are thus labelled ‘`L1`’ and ‘`L2`’ here.

The indicator’s intercepts are also provided a label, and are specified as by regressing the variable on 1. For the first indicator the syntax reads:

```
F ~ Int_f*1
```

In the model with strong invariance the intercepts and factor loadings are not moderated by culture. The correlation between the two common factors is moderated. This is specified as follows:

```
Innov ~~ Phi21*Adapt

Phi21 == Phi21_west + Phi21_diff*data.NonWestern
```

The ‘==’ operator specifies how the parameters on the left side are a function of the parameters at the right side. The right-hand side here contains the part ‘`data.NonWestern`’. This refers to a definition variable--the specific study’s value on the variable ‘`NonWestern`’ in `df$data`. The parameters `Phi21_west` and `Phi21_diff` thus represent the intercept of the correlation between `Innov` and `Adapt`, and the regression coefficient of ‘`NonWestern`’ on this correlation.

The factor variances are fixed to 1 in one group, and freely estimated in the other. This is also specified using labels and the ‘==’ operator, by fixing the intercepts of the variances to 1:

```
Innov ~~ Phi11*Innov

Adapt ~~ Phi22*Adapt

Phi11 == 1 + Phi11_diff*data.NonWestern

Phi22 == 1 + Phi22_diff*data.NonWestern
```

A similar structure is used to fix the factor means to 0 in in the Western studies, and to freely estimate the factor mean of the non-Western studies:

```
Innov ~ k1*1

Adapt ~ k2*1

k1 == 0 + Innov_meandiff*data.NonWestern

k2 == 0 + Adapt_meandiff*data.NonWestern
```

Lastly, the residual variances are all moderated by culture. For the first indicator the syntax is:

```
F ~~ ResVar_F*F

ResVar_F == ResVar_F_west + F_diff*data.NonWestern
```

The syntax is stored in the R-object '`model`'. The complete syntax for specifying the model with strong factorial invariance across groups of studies is:

```
model <- "## Factor loadings (not moderated)

          Innov =~ L1*F + L2*O

          Adapt =~ L3*T + L4*E + L5*R


          ## intercepts (not moderated)

          F ~ Int_f*1

          O ~ Int_o*1

          T ~ Int_t*1

          E ~ Int_e*1

          R ~ Int_r*1
```

```
## Factor correlation (moderated)

Innov ~~ Phi21*Adapt

Phi21 == Phi21_west + Phi21_diff*data.NonWestern



## Factor variances (moderated, fixed at 1 for Western)

Innov ~~ Phi11*Innov

Adapt ~~ Phi22*Adapt

Phi11 == 1 + Phi11_diff*data.NonWestern

Phi22 == 1 + Phi22_diff*data.NonWestern



## Factor means (moderated, fixed at 0 for Western)

Innov ~ k1*1

Adapt ~ k2*1

k1 == 0 + Innov_meandiff*data.NonWestern

k2 == 0 + Adapt_meandiff*data.NonWestern



## Residual variances (moderated)

F ~~ ResVar_F*F

ResVar_F == ResVar_F_west + F_diff*data.NonWestern

O ~~ ResVar_O*O

ResVar_O == ResVar_O_west + O_diff*data.NonWestern
```

```
T ~~ ResVar_T*T

ResVar_T == ResVar_T_west + T_diff*data.NonWestern

E ~~ ResVar_E*E

ResVar_E == ResVar_E_west + E_diff*data.NonWestern

R ~~ ResVar_R*R

ResVar_R == ResVar_R_west + R_diff*data.NonWestern"
```

The '`lavaan2RAM()`' function is used to create the model matrices as used in OpenMx (using the RAM-formulation) based on the lavaan syntax. It uses the `obs.variables` argument to determine the ordering of the variables in the model matrices. It is therefore very important to specify the names of the observed variables in the same order as what was used in the observed data. The object '`varnames`' here contains a character vector with the observed variable names (F, O, T, E, and R) The argument '`std.lv = FALSE`' is needed to freely estimate the factor variances in the non-Western studies. Setting this argument to `TRUE` (the default) will fix all common factor variances to 1.

```
RAM <- lavaan2RAM(model, obs.variables = varnames, std.lv = FALSE)
```

The `osmasem2()` function fits the specified model ('`RAM`') to the data '`df`'.

```
fit_dummy_strong <- osmasem2(model.name="Strong invariance",
RAM=RAM, data=df, cor.analysis=FALSE, mean.analysis=TRUE,
replace.constraints = TRUE)
```

The first argument of this function can be used to provide a name to the model. Next, it needs the RAM-object of the model, and the data. Since we are analyzing covariances and not correlations, we set `cor.analysis` to `FALSE`, for analyzing the mean structure we set `mean.analysis` to `TRUE`. The `replace.cons`traints argument should be set to `TRUE` as soon as there are parameters specified to be a function of other parameters using the '==' operator.

If the analysis does not lead to a converged solution, it may be needed to rerun the model using the `rerun()` function:

```
fit_dummy_strong <- rerun(fit_dummy_strong)
```

Lastly, the results can be viewed by asking the summary of the R-object containing the output:

```
summary(fit_dummy_strong)
```
